# Piezoresistive Strain Sensors Made from Carbon Nanotubes Based Polymer Nanocomposites

**DOI:** 10.3390/s111110691

**Published:** 2011-11-11

**Authors:** Alamusi, Ning Hu, Hisao Fukunaga, Satoshi Atobe, Yaolu Liu, Jinhua Li

**Affiliations:** 1 Department of Mechanical Engineering, Chiba University, Yayoi-cho 1-33, Inage-ku, Chiba 263-8522, Japan; E-Mails: alamusi@chiba-u.jp (A.); liuyaolu@graduate.chiba-u.jp (Y.L.); lijinhua0312@yahoo.co.jp (J.L.); 2 Department of Aerospace Engineering, Tohoku University, Aramaki-Aza-Aoba 6-6-01, Aoba-ku, Sendai 980-8579, Japan; E-Mails: fukunaga@ssl.mech.tohoku.ac.jp (H.F.); atobe@ssl.mech.tohoku.ac.jp (S.A.)

**Keywords:** carbon nanotubes, nanocomposites, strain sensor, piezoresistivity

## Abstract

In recent years, nanocomposites based on various nano-scale carbon fillers, such as carbon nanotubes (CNTs), are increasingly being thought of as a realistic alternative to conventional smart materials, largely due to their superior electrical properties. Great interest has been generated in building highly sensitive strain sensors with these new nanocomposites. This article reviews the recent significant developments in the field of highly sensitive strain sensors made from CNT/polymer nanocomposites. We focus on the following two topics: electrical conductivity and piezoresistivity of CNT/polymer nanocomposites, and the relationship between them by considering the internal conductive network formed by CNTs, tunneling effect, aspect ratio and piezoresistivity of CNTs themselves, *etc*. Many recent experimental, theoretical and numerical studies in this field are described in detail to uncover the working mechanisms of this new type of strain sensors and to demonstrate some possible key factors for improving the sensor sensitivity.

## 1. Introduction

Various nano-scale carbon fillers of high aspect ratio, such as carbon nanotubes (CNTs) and vapor growth carbon fibers (VGCFs), possess excellent mechanical properties and electrical conductivities. Besides applications of a single CNT in various nanoelectronic applications, such as probes [1] or oscillators [2], CNTs are also ideal structural components candidates in various composites and functional composites due to their mechanical reinforcement effects [3–7]. In functional composites, for instance, it is possible to produce conductive polymer nanocomposites with a small amount of CNTs which are dispersed in insulating polymers. This new type of electrically conductive CNT/polymer nanocomposite can be applied to various fields, such as piezoresistive or resistance-type strain sensors of high sensitivity, electromagnetic interference materials, *etc*. In the field of resistance-type strain sensors made from these new materials, for instance, it has been confirmed that the conductivity of a single-walled carbon nanotube (SWNT) could be dramatically changed by introduction of strain using atomic force microscopy (AFM), as a consequence of the band-gap and structural changes under the effect of mechanical strain [8]. Due to the piezoresistivity property of CNTs themselves and other working mechanisms described later, it was predicted that integrating CNTs into polymers would open up a whole range of smart structure applications [9,10]. In particular, great interest has recently been aroused in building strain sensors with CNTs [11–34], carbon nano-blacks [23] and graphene [35], although in this article, we mainly focus on CNT/polymer nanocomposite strain sensors. This type of strain sensors with outstanding static and low-frequency dynamic responses is very hopeful for its implementation on various structures to carry out structural health or integrity monitoring tasks, e.g., dynamic contact or impact events monitoring [36–38], and various damages, e.g., delamination in laminates [39–43].

Generally, this new type of strain sensors can be employed practically through two main approaches. On one hand, CNTs are generally Raman active, and can be blended with a polymer to make a strain sensor provided a relationship between mechanical strain and Raman spectrum shift can be calibrated [11,14]. Obviously, implementation of complex equipment in this technique remains a technical challenge, especially for potential field applications. Alternatively, macro-scale resistance-type strain sensors, e.g., with dimensions of mm or cm, have been increasingly used to measure static and low-frequency dynamic strains on the surfaces of a structure. To this end, two types of strain sensors have been developed, *i.e.*, SWNT buckypaper sensors [12,13,15] and sensors made from various CNT/polymer nanocomposites, e.g., SWNT or multi-walled carbon nanotube (MWNT) or carbon nanofibers were widely used [16–34]. Except for [16], which added SWNTs and MWNTs into PVDF, *i.e.*, a piezoelectric polymer to fabricate a piezoelectric-type strain sensor, most studies [17–34] have focused on resistance-type strain sensors. A common feature of these resistance-type nanocomposite sensors, which is of the most importance, is that as compared to conventional strain sensors, e.g., strain gauges, higher sensitivity has been observed in these novel sensors, at least at a macro-scale [15,19,21,27,28,30,34]. This advantage can hopefully lead to useful applications, especially, large-scale neuron sensor networks on various structures working as human skins. In spite of the above-mentioned promising results and advances, fundamental understanding of piezoresistivity behavior in CNT/polymer nanocomposite is still lacking, largely due to the less effort expended on such studies, except for those [25,27–29], being put into theoretical and numerical investigations on the piezoresistivity behavior in these nanocomposites.

This article reviews the recent research outcomes concerning resistance-type strain sensors made from CNT/polymer nanocomposites. Here, we focus our attention mainly on the piezoresistivity of these nanocomposite strain sensors since this way is more practical and cheaper compared with measurements of Raman spectrum shift of these nanocomposites under strain. The CNT buckypaper sensors [15,33] are also interesting. However, they do not fall within the primary scope of this article. Basically, the working mechanisms of CNT buckypaper sensors, which have very small fracture strain [15] and poor stability that limit their wide applications, should be similar to those of CNT/polymer nanocomposites. To explain the piezoresistivity behavior of strain sensors made from CNT-filled polymer nanocomposites, it is crucial, at the first stage, to comprehensively understand the electrical conductivity phenomena of these nanocomposites containing a conductive network formed by CNTs. Therefore, this article is organized as follows. Section 2 gives a detailed and comprehensive description of the electrical conductivity of CNT/polymer nanocomposites by referring to recent research outcomes in this field. Especially, the process of formation of a conductive network by CNTs in thermosetting polymer matrices is described in detail. In Section 3, we describe the piezoresistivity behavior of CNT/polymer nanocomposites from an experimental and numerical point of view. Sensor working mechanisms are discussed in detail in this section by explaining their relationship with the electrical conductivity. Section 4 provides some important conclusions, which include some possible approaches to improve sensor sensitivity.

## 2. Electrical Conductivity of CNFs/Polymer Nanocomposites

In recent years, much attention has been paid to the fabrication of nanocomposites with use of various CNTs in polymer materials to harness the exceptional electrical properties of CNTs. In particular, polymers with the incorporation of CNTs show great potential for electronic device applications, such as organic field emitting displays, photovoltaic cells, highly sensitive strain sensors, electromagnetic interference materials, *etc*. Generally, different electrical properties of nanocomposites are employed for these applications. For instance, for the application of strain sensors, the direct current (DC) properties of nanocomposites are needed. Meanwhile, for the application of electromagnetic interference materials, the alternate current (AC) properties of nanocomposites are necessary. In the past decade, numerous experimental studies on the electrical properties of nanocomposites made from insulating polymers filled by CNTs have been carried out [44–66]. However, in this field, numerical and theoretical studies, e.g., [49,63,67] are very limited to date.

**Figure 1.**
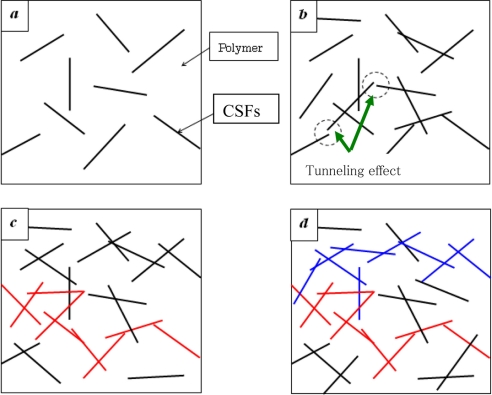
Percolation process in conductive composites.

**Figure 2.**
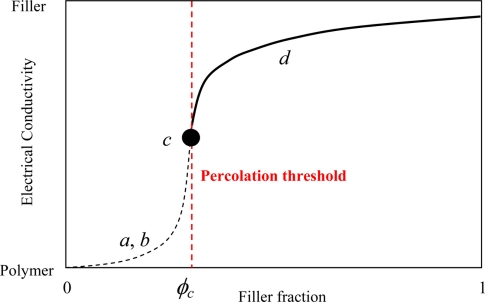
Electrical conductivity of conductive composites as a function of filler fraction, where “*a*, *b*, *c*, *d*” denote the different states in Figure 1.

In this section, we focus on reviewing of DC properties of nanocomposites. Generally, by gradually filling some traditional conductive filler particles, e.g., carbon short fibers (CSFs), into insulating polymers, the variation of electrical conductivity of composites can be divided into three stages, as shown in Figures 1 and 2. In the first stage, the electrical conductivity is very low since there are only a few CSFs, as shown in Figure 1(a,b). The electrical conductivity of composites is close to that of the polymer matrices, as shown in Figure 2. However, it should be noted that, in Figure 1(b), some large clusters connected by CSFs are gradually formed. There are some CSFs which are close to each other. Therefore, in this state “*b*”, the electrical conductivity of composites increases gradually due to tunneling effects among those neighboring CSFs, although there is no complete conductive path formed by contacting CSFs. As explained later this state “*b*” is very important for the piezoresistivity of nanocomposites. In the second stage, as the amount of CSFs increases, the first complete electrically-conductive path connected by some law as is formed as shown in [Figure 1(c), red path].

In this second stage, the electrical conductivity of composites increases remarkably following a percolation power law as shown in Figure 2. This process is termed the percolation process. The volume fraction of filler particles at this stage is called as the percolation threshold, *i.e.*, *ϕ_c_* in Figure 2. In the final stage, with the further addition of filler particles into the polymer matrix, a lot of electrically-conductive paths, which forms a conductive network, can be constructed, as shown in Figure 1(d), and the electrical conductivity of composites further increases gradually, until leveling off at a constant, which is lower than that of the element or filler of conductive network in Figure 2. From the previously published experimental results, it was found that the electrical behavior of nanocomposites using CNTs as conductive filler particles in polymer matrices, e.g., [48–60,63,66] follows the similar percolation phenomenon to that stated above for traditional conductive filler particles, e.g., CSFs. Here we only briefly review some limited references on the electrical percolation phenomenon of CNT/polymer nanocomposites, and one can refer to the outstanding review article by Bauhofer and Kovacs [66] for more detailed information.

In the experimental studies in this field, currently, melt mixing compounding [44–47], curing/*in situ* polymerization [48–60,63] and coagulation [61,62] are widely used to prepare this kind of nanocomposites using CNTs.

Depending on the type of polymer matrix and processing technology as well as the type of CNT materials used, percolation thresholds ranging from less than 1.0% to over 10.0 wt.% of CNTs loading have been observed experimentally [54,66]. For example, for SWNTs, Nogales *et al*. [48] applied *in situ* polycondensation reaction to prepare SWNT/PBT nanocomposites and achieved an electrical percolation threshold as low as 0.2 wt.% of SWNTs loading. Ounaies *et al*. [49] have investigated the electrical properties of SWNTs reinforced polyimide (CP2) composites. The obtained conductivity obeys a percolation-like power law with a low percolation threshold of around 0.1 wt.%. The bundling phenomenon of SWNTs within the matrix has been identified in experimental analysis. Park *et al*. [50] have shown that it is possible to control the electrical properties of SWNT/polymer composites through the techniques of alignments of SWNTs. Kymakis *et al*. [51] studied the electrical properties of SWNTs filled in the soluble polymer poly(3-octylthiophene) (P3OT). The reported percolation threshold is around 11.0 wt.%. In their later work [52], purified SWNTs were used, which lead to a much lower percolation threshold of around 4 wt.%.

For MWNTs, Sandler *et al*. [53] have employed MWNTs with an epoxy polymer based on bisphenol-A resin and an aromatic hardener, and they got a lower percolation threshold at around 0.04 wt.%. The formation of aggregates was also identified. Sandler *et al*. [54] reported the lowest percolation threshold up to the present date, *i.e.*, 0.0025 wt.% using MWNTs. To obtain a low percolation threshold, using MWNTs and epoxy, Martin *et al*. [55] investigated the influence of process parameters employed in an *in situ* polymerization fabrication process, such as stirring rate, resin temperatures and curing temperatures. It was found that the electrical properties of nanocomposites strongly depend on the choice of these parameters. Using an *in situ* polymerization process, MWNT/polymer nanocomposites were prepared in [56–58], and the obtained percolation thresholds were found to be lower than 1.0 wt.%. Hu *et al*. [62] prepared MWNT/PET nanocomposites by means of a coagulation process. Uniform dispersion of MWNTs throughout PET matrix was confirmed by transmission electron microscopy (TEM) and scanning electron microscopy (SEM). The obtained percolation threshold was around 0.9 wt.%. As mentioned above, although a lot of experimental studies have been performed recently, except for [55], there is little literature covering the detailed influences of various factors in fabrication process on the electrical properties of CNT/polymer nanocomposites using *in situ* polymerization methods. For this reason, the present authors prepared the MWNT/epoxy nanocomposites and obtained a low percolation threshold of 0.1 wt.% [63]. The effects of curing process, mixing speed, mixing time, addition of ethanol, timing of hardener addition, *etc.*, in the fabrication process on the electrical properties of nanocomposites have been investigated in detail [63]. It was found that the curing temperature and the mixing conditions are key factors in the fabrication process, which influence the formation of conducting network significantly. Therefore, careful design of these factors in the fabrication process is required to achieve high electrical performances of nanocomposites [63]. A three roll milling technique was also used to improve the dispersion of CNTs [64] to get the highly conductive CNT/epoxy nanocomposites. All of above experimental studies have provided the comparatively stable conductivity at high CNT loadings (state “*d*” in Figures 1 and 2), which ranges from several S/m to several hundreds of S/m (e.g., [63,64]).

Generally, there are two key issues being addressed in many previous experimental studies [55,63]: dispersion of CNTs in a polymer matrix and interaction between CNTs and polymer. For the first issue, due to the high surface-to-mass ratio of CNTs, molecular scale forces and interactions should be considered among CNTs. van der Waals forces usually promote flocculation of CNTs, whilst electrostatic charges or steric effects lead to a stabilization of the dispersion through repulsive forces [49,55,63]. As a consequence, by considering the nature of a percolating network formed by very fine filler particles, e.g., CNTs, the balance of the two factors of reverse effects outlined above should be taken into account. For the second issue, the fact that the nanotubes in the composites were coated or encapsulated with a thin insulating polymer layer was identified for SWNTs [52] and MWNTs [62]. This encapsulation acts as a barrier to the electrical charge transfer between nanotubes [52]. For the dispersion of CNTs and encapsulation of nanotubes by polymer chains, there are three very important conclusions obtained in [63] for thermosetting resins, which are tightly related to the subsequent piezoresistivity issue in the following sections:

- It was found that a high temperature in the curing process can increase the electrical conductivity of nanocomposites since the macroscopic conducting network may be formed more easily by enhancing mobility of CNTs in the resulted accelerated diffusion process;
- The effects of mixing speed and mixing time are complex, however, a mixing process with modest shear forces and short mixing time, which is helpful to the formation of macroscopic conducting networks of MWNTs, is certainly enough since there is usually no significant aggregate of MWNTs as identified in many previous studies [55,63]. Too high shear forces and too long mixing time may break up the networks of MWNTs. This result implies that an optimal mixing process exists to avoid both over-dispersion and intensive aggregation of MWNTs for enhancing the electrical conductivity of nanocomposites at low volume fractions of MWNTs. For SWNTs, the situation may be different due to much higher adhesive forces among SWNTs caused by van der Waals interactions. In this case, much higher mixing speed and longer mixing time may be needed;
- The encapsulation of nanotubes by polymer chains is very complex, however, it may be helpful to use a procedure in which the mixture of epoxy and hardener are first prepared with the subsequent addition of MWNTs.

Compared with the above huge amount of experimental studies, unfortunately, there have been very few systematic theoretical or numerical studies aimed at comprehensively understanding the electrical characteristics of CNT/polymer nanocomposites at and after the percolation threshold. For instance, the percolation threshold value was determined by a numerical model [25,49,63] with randomly distributed CNTs in a polymer and by an empirical formula from the extruded volume approach based on the statistical percolation theory [68]. For the electrical conductivity, a micromechanics average method based on representative volume element (RVE) model was developed to assess the effects of electron hopping and the formation of conductive networks on the electrical conductivity of CNT/polymer nanocomposites [67]. In fact, for some electronic composites with some traditional conductive filler particles, e.g., CSFs or carbon flakes, there have been some theoretical or numerical studies based on the traditional statistical percolation model [68–70], especially for predictions of percolation threshold. It is therefore natural to ask if the statistical percolation model is still valid to describe the electrical behaviors of the nanocomposites with such fine filler particles as CNTs. The work of the present authors [71] may partially answer this question. In [71], for an insulating polymer with random distribution of CNTs, firstly, based on the statistical percolation model, a three dimensional (3D) numerical model with two stages for investigating the electrical properties of nanocomposites at and after the percolation threshold was developed. In the first stage, the percolation threshold was predicted at the volume fraction of CNTs when the first complete electrically-conductive path connected by some CNTs is formed in the polymer matrix. In the second stage, a 3D resistor network model was constructed to predict the macroscopic electrical conductivity of nanocomposites after the percolation threshold. This model demonstrates remarkable success in capturing the main features of electrical behaviors of nanocomposites. Influences of various factors, such as curved shapes of CNTs, aggregation of CNTs and tunneling effect among CNTs on the electrical properties of nanocomposites have been studied. Then, the verified numerical model was employed to construct a simple and reliable empirical percolation theory.

The experimental results [63] obtained by the present authors plus some other previous experimental results [56,57] using the same MWNTs have been employed to validate the proposed numerical model. In this article, we mainly describe the results obtained in [71] for improving the understanding of the percolating electrical conductive phenomenon of CNT/polymer nanocomposites.

**Figure 3.**
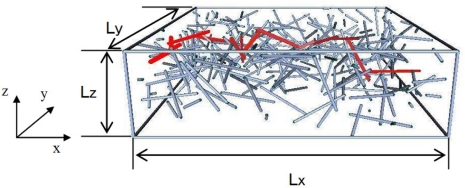
Schematic view of a representative 3D element with randomly dispersed CNTs.

**Figure 4.**
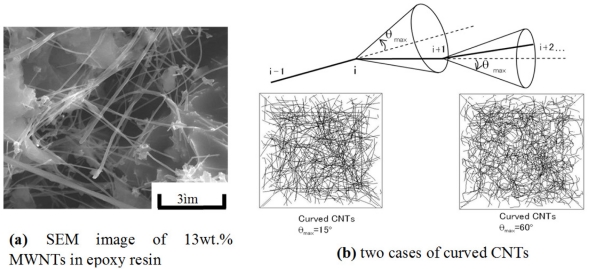
SEM image and numerical model of curved CNTs.

Firstly, to predict the percolation threshold of the nanocomposites with CNTs, as shown in Figure 3, a 3D representative element with a random distribution of CNTs was used [71]. To reduce the computational cost in the Monte-Carlo procedure used, the CNTs were considered as capped cylinders of length *L* and diameter *D*. These cylinders with random orientations were distributed in a cube, *i.e.*, a unit cell. The union/finding algorithm [72] was adopted to detect the first complete conductive path spanning the 3D element (red CNTs in Figure 3), and the percolation threshold could then be determined from the total volume of capped cylinder CNTs and the volume of the representative element. As shown in Figure 4, the curved CNTs were also modeled by proposing a method with a representative parameters *θ*_max_, in which a whole CNT was divided into several segments.

**Figure 5.**
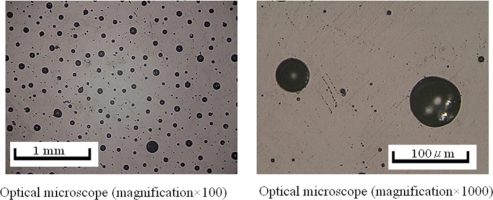

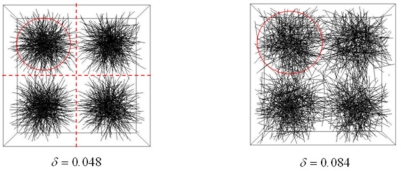
Experimental images of CNT aggregates and numerical model of agglomerated CNTs with a normal distribution.

Moreover, as shown in Figure 5, the aggregates were modeled by proposing a method with a representative parameter *δ*, whose small value denotes an intensively aggregated state.

**Figure 6.**
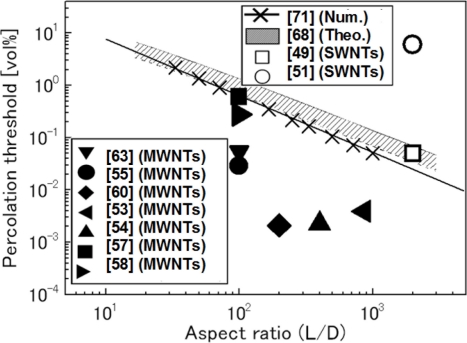
Comparison of numerical percolation threshold and experimental results for straight CNTs.

The influences of both curved shape and aggregation on the percolation threshold and electrical conductivity as stated later were comprehensively explored. For the percolation threshold, the comparison between the numerical results [71] with the theoretical one [68] and some experimental results [49,51] for SWNTs, and [53–55,57,58,60,63] for MWNTs is shown in Figure 6 where *L*/*D* is the aspect ratio of CNTs with the length *L* and the diameter *D*, respectively. The numerical results [71] agree with the theoretical one [68] very well. It is interesting to note that the experimental percolation thresholds of SWNT/polymer nanocomposites are higher than the numerical prediction. For those high experimental percolation thresholds of SWNTs, as explained in [51], one possible reason is the impurity of SWNTs used, *i.e.*, the lack of uniformity of electrical conductance in SWNTs. Another may be attributed to the difficulty in uniform dispersion of SWNTs due to very high absorption energy of SWNTs, as demonstrated in [49]. On the contrary, the experimental percolation thresholds corresponding to MWNTs are lower than the numerical and theoretical predictions in Figure 6. This may be explained by easy dispersion of MWNTs in the polymer matrix, and easy formation of a macroscopic conducting network due to small-scale chain-like aggregates of MWNTs, as pointed out in [63].

**Figure 7.**
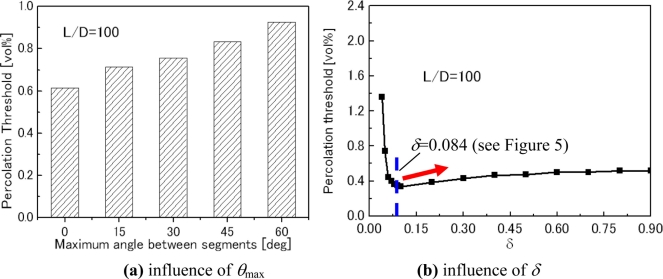
Influences of curved shape (*θ*_max_) and aggregate severity (*δ*).

The influences of the curved shape of CNTs denoted by *θ*_max_ and the aggregate severity denoted by *δ* are shown in Figure 7. For the curved CNTs [Figure 7(a)], the percolation threshold increases gradually with *θ_max_*, indicating that the formation of the first conductive path becomes more difficult compared to the straight CNTs. The influence of aggregates on the percolation threshold is shown in Figure 7(b).

It is clear that a very high concentration of aggregates (*i.e.*, very small *δ*) results in high percolation thresholds. However, when *δ* is larger than a critical value, *i.e.*, 0.084 in Figure 7(b), the aggregates have no obvious influence on the percolation threshold of nanocomposites. A very interesting phenomenon is that the percolation threshold is the lowest one when *δ* = 0.084. As the dispersion state becomes better, *i.e.*, increase of *δ*, the percolation threshold unexpectedly increases [red arrow in Figure 7(b)]. The reason may be from a lightly aggregated state, e.g., small-scale chain-like aggregates of CNTs, being helpful for forming the first conductive path in a matrix. A perfect dispersion state of CNTs leads to individual CNTs separated in the matrix, and therefore, a higher percolation threshold. It should be noted that a recent experimental study [65] confirms this numerical result. Aguilar *et al.* [65] identified that the percolation thresholds were 0.11% wt.% and 0.068% wt.% for the uniformly dispersed and agglomerated films of MWNT/polymer nanocomposite, respectively, which indicates that the lightly agglomerated state of CNTs may be helpful to decrease the percolation threshold. This point can also be used to explain why the experimental results of MWNTs are lower than the numerical and theoretical ones based on the assumption of complete and ideal random distribution or dispersion state of CNTs. For this issue, in fact, the observation of two percolation thresholds, *i.e.*, statistical percolation and kinetic percolation, in the same MWNT/epoxy system was reported in [66]. The reason for that some experimental percolation thresholds (kinetic percolation) are significantly lower than the theoretical statistical percolation threshold, is attributed to kinetic percolation which allows for particle movement and re-aggregation [66]. Therefore, aggregation is a complex problem. It is important to select a proper dispersion process to avoid both intensive aggregates and the ideal or perfect dispersion state of CNTs for obtaining the lowest percolation threshold. As experimental confirmed in [65], this statement is also valid for obtaining a higher electrical conductivity of CNT/polymer nanocomposites in the stage after the percolation threshold [Figure 1(d)] although it is not as obvious as to the percolation threshold.

Finally, based on the results of Figure 6, the relationship between the percolation threshold and *L*/*D* of CNTs can be established as: *ϕ_c_* = (*L*/*D*)^−1.1 ± 0.03^ as summarized in [71]. This formula is much simpler than other empirical models for prediction of the percolation threshold, e.g., the model in [68]. It should be noted that this formula is only valid for the fillers with high aspect ratio (e.g., over 20).

**Figure 8.**
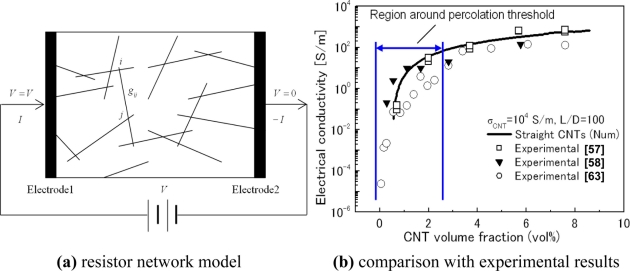
Resistor network model and comparison between numerical results and experimental ones.

For the electrical conductivity after the percolation threshold [71], as shown in Figure 8, a 3D resistor network model [73,74] for predicting electrical conductivities in some traditional composites has been adopted, provided the nanocomposite microstructures can be numerically simulated. The numerically obtained electrical conductivity was compared with three experimental data [57,58,63] which employed the same MWNTs of the aspect ratio as 100. The conductivity of CNTs was taken as 10^4^ S/m since, generally, *σ_CNT_* for MWNTs ranges from 5 × 10^3^ to 5 × 10^6^ S/m as reported in [75,76]. It can be found from Figure 8(b) that the numerical results [71] agree with the experimental ones very well, which validates the effectiveness of the proposed numerical model.

In [71], the effects of curved shape and conductivity of CNT filler on the electrical behavior of nanocomposites were also investigated using the above 3D resistor network model. The obtained conclusions are summarized in the following. For straight CNTs, the electrical conductivity of nanocomposites is proportional to the electrical conductivity and the aspect ratio of CNTs. A higher CNT aspect ratio also leads to a lower percolation threshold (see Figure 6). On the other hand, the curved shape of CNTs leads to a lower electrical conductivity but its effect is limited. However, the influence of aggregates on the electrical conductivity of nanocomposites is very significant. The electrical conductivity decreases with *δ*. For very small *δ*, there are strong discontinuities in the results.

In [71], the traditional percolation theory, e.g., [77], was also discussed based on the numerical simulations. According to the traditional percolation theory [77], the electrical conductivity of electronic composites can be predicted as: *σ_com_* = *σ*_0_ (*ϕ* – *ϕ_c_*)*^t^* for *ϕ* > *ϕ_c_*, where *t* is the critical exponent, *ϕ* is the volume fraction of filler, *ϕ_c_* is the percolation threshold, and *σ*_0_ is a parameter basically depending on the electrical conductivity of filler in traditional percolation theories. Usually, *ϕ_c_*, *t* and *σ*_0_ can be determined experimentally. By considering an ideal random distribution of straight CNTs in a polymer matrix, the average *t* was identified as 1.8 ± 0.05 by least-squares fitting from the numerical results using the above traditional percolation theory. The effect of aspect ratio of CNTs on *t* was investigated. As obtained in [71], *t* (the slope of curves) is not sensitive to the aspect ratio. As noted in [78], *t* is only dependent on the dimensionality of the system. Moreover, the influence of the curved shape of CNTs on *t* is not significant. When *ϕ* − *ϕ_c_* is very small, the slope of the curves corresponding to the curved CNTs is almost the same as the straight CNTs (*t* = 1.8). However, when *ϕ* − *ϕ_c_* is greater than a certain limit, e.g., 0.01 in [71], the curved CNTs leads to a slightly lower *t* (*t* = 1.65). It means that over a certain volume fraction of CNTs, the formation of conductive network by the curved CNTs is more difficult as compared to the straight CNTs. Moreover, it was found that the aggregates lead to a lower *t*.

Finally, based on the numerical data, the electrical conductivity of CNT/polymer nanocomposites has been obtained as follows in [71]: *σ_com_* = *σ_CNT_* ·10^0.85{log(*L*/*D*)−1}^·{*ϕ* – *ϕ_c_*}*^t^*. It is worthwhile exploring the effects of the aspect ratio of CNTs (*L*/*D*) on *σ*_0_ in the above equation by comparing with the traditional percolation theory. From the numerical data [71], it is very interesting to note that *σ*_0_ depends not only on *σ_CNT_* (electrical conductivity of CNTs), but also the *L*/*D* as well. This can be regarded as a new finding since *σ*_0_ has been generally considered to be dependent only on the electrical conductivity of filler in all existing traditional percolation theories, especially for low filler volume fractions [77,78]. This finding, *i.e.*, the effect of aspect ratio on the electrical conductivity of CNT/polymer nanocomposites, has been experimentally verified in a latter research [64]. Besides its application to CNT/polymer nanocomposites, the above formulation [71] has also been verified by experimental data of composites with CSFs and nanocomposites with nanofibers.

## 3. Piezoresistivity of CNFs/Polymer Nanocomposites

### 3.1. Experimental Investigations

After understanding the electrically percolating phenomenon of an insulating matrix filled by conductive CNTs, we mainly review the recent outcomes on the development of strain sensors by using the piezoresistivity of CNT/polymer nanocomposites although the results of carbon nano-blacks and graphene based nanocomposite sensors are also briefly described. Here, the piezoresistivity is defined as: Δ*R* / *R*_0_, where *R*_0_ is the initial electrical resistance and Δ*R* is the electrical resistance change at a specified strain level. Firstly, the experimental studies are reviewed in this section.

To date, there have been a lot of experimental studies for resistance-type nanocomposite strain sensors, e.g., [12,13,15,17–24,26–28,30–32,34]. In the nanocomposite sensors, the widely used nano-scale carbon filler particles are SWNTs [12,13,15,20], MWNTs [15,17–19,21–24,27,30–32], carbon nanofibers [24,28,34], e.g., vapor growth carbon nanofibers (VGCF) [28,34], carbon nano-blacks [23] and graphene [35]. The influence of the type of nano-scale carbon fillers on the sensor piezoresistivity may be very significant from the following several aspects: size, shape, aspect ratio and electrical conductivity of filler particles. These factors will be discussed in detail later. For insulating polymer matrices, there are traditional epoxy resin [21–23,27,30], flexible epoxy [28,34], PMMA [15,19], PC [17], PEO [18], PE [20], PU [24], PP [26], PSF [31,32], *etc*. Basically, the influence of polymer type on the piezoresistivity may be comparatively small. The most significant influence from the polymer type may be from its viscosity which determines the characteristics of mixing process and dispersion state since, basically, the thermosetting plastics and thermoplastics have the different viscosities and fabrication processes. Another influence of the polymer type may be in tunneling effect, which will be stated later. Most of the previous studies employed a four-point probe measurement technique, e.g., in [12,13,20,21,27,30], or a two-point probe measurement technique, e.g., in [19,28,34]. Besides static responses of nanocomposite sensors, the dynamic responses were also measured in some studies, e.g., [15,17,19,20,30]. Moreover, many studies focused on the sensor behaviors under tensile strains, e.g., [18,21,23,26,28,32], however, the sensor behaviors under compressive strains were also investigated in some studies, e.g., [12,20,27,30]. Here, it should be noted that the thermal stability of CNT/polymer sensors should be an important issue since the electrical resistivity of CNTs and epoxy polymer properties may depend on environment temperatures. However, this issue does not belong to the content of this article, and all of stated experimental data in the following were obtained in room temperature.

Due to a variety of nano-scale carbon filler particles, polymer matrices, fabrication processes and measurement techniques, the obtained sensor piezoresistive behaviors are also diversified. For the sensor piezoresistive behaviors, some most important experimental evidences obtained in the above references are summarized as follows:

- For SWNTs used in some previous studies [12,13,15], or a special type of MWNT, *i.e.*, LMWNT-10 of a diameter being smaller than 10 nm in [30], which is similar to a SWNT, the linear piezoresistivity responses of nanocomposite sensors in static tests have been identified within the different strain ranges. For example, there were small strain ranges, e.g., ±200 με [12,13], ±1,300 με [15], and comparatively large strain ranges, e.g., ±6,000 με [30].
- For MWNTs [17–19,21–24,27,30,32] or carbon nanofibers (e.g.,VGCFs) [24,28,34] whose diameter is comparatively large and whose shape is comparatively straight, a linear piezoresistivity for low strains followed by a nonlinear piezoresistivity at large strains has been identified in most of previous studies, e.g., [18,19,21–24,26–28,30,32,34], and [31] for samples without applied AC voltages to adjust the alignment of MWNTs. An interesting phenomenon in [31] is that after adjusting the alignment of MWNTs using applied AC voltages, the linear piezoresistivity has been obtained. Moreover, it should be noted for carbon nano-blacks in [23], the nonlinear piezoresistivity has also been identified, which is more obvious compared with MWNT nanocomposites. Only within a low strain value (1.0%), the piezoresistive response of carbon nano-blacks or MWNT nanocomposites [23] can be approximated fairly well with a linear function. For graphene/epoxy nanocomposite sensors in [35], an approximate linear behavior within ±1,000 με was identified.
- For the relationship between the piezoresistivity (*i.e.*, sensor sensitivity or gauge factor) and the CNT loading, in most of previous studies for MWNTs and carbon nanofibers (e.g., VGCFs) [18,19,21–24,26–28,30,31,34], and for SWNTs [15], it was found that with the decrease of CNT loading, the piezoresistivity or sensor sensitivity increases monotonically. In [32], gauge factors were measured for films with 0.2∼1.0% MWNT weight loadings. The best piezoresistive capabilities were found for films with MWNT loadings as low as 0.5% weight loadings. Further increments in MWNT loading did not produce an appreciable increment in the film sensor sensitivity. There have been very few references, e.g., [20], which reported that the increase in CNT loading allows the SWNT-PSS/PVA film sensor to be more sensitive to strain. This phenomenon was explained due to creation of more nanotube-to-nanotube junctions [20]. In our previous work [30], by using a special type of MWNT, *i.e.*, LMWNT-10 of a diameter being smaller than 10 nm, which can be considered to be similar to a SWNT, we have also identified that there is no direct relationship between the CNT loading and the sensor gauge factor. Basically, the above observed results for piezoresistivity were obtained in the tension state.
- For the sensor piezoresistive behaviors in compressive strains, there have been very few reported results. For SWNTs and very small measured strain ranges, e.g., ±200 με [12,13], ±1,300 με [15], it was found that the piezoresistivity behaviors in both tensile and compressive strains are linear and anti-symmetric about the zero strain point. For comparatively large strain ranges, e.g., ±6,000 με, similar behavior has also been identified for LMWNT-10 [30] being similar to a SWNT. For MWNTs of comparatively large diameter and straight shape, it was identified that the above conclusion is only valid for a very small strain range, *i.e.*, ±1,000 με in [27]. When exceeding this range, the sensor behavior in the compressive side is completely different with that in the tensile side. With the increase of compressive strain, the sensitivity of a sensor decreases and finally saturates [27]. In fact, this difference between the tensile behavior and compressive behavior was also observed in the strain sensors made from the epoxy polymer and traditional CSFs [79] whose size is much larger than that of CNTs discussed in the present article. For the measured gauge factors defined as: (Δ*R* / *R*_0_) / ε, there is also a great variety in the above previous studies. However, most of the previous data, except for [20] for SWNTs and [32] for MWNTs, reveal that the obtained sensor gauge factors are higher than that of traditional metal-foil strain gauges whose gauge factor is approximately equal to 2. Basically, due to the possible nonlinear piezoresistivity, especially for the sensors using MWNTs and carbon nanofibers, strictly, the gauge factor should be defined according to a specified strain level, e.g., in [21,27,30]. For SWNTs, there are only a few data. In [15], the highest gauge factor is around 5.0 for 0.5 wt.% of SWNTs within the strain range ±1,300 με for obtaining the linear sensor response. In [20] using SWNTs, the strain sensitivities between 0.1 and 1.8 have been achieved, which are lower than that of traditional metal-foil strain gauges. In [30], when using LMWNT-10 like a SWNT, the obtained sensor gauge factor ranges from 3.8∼5.8 for both tension and compression states. For MWNTs and carbon nanofibers, the obtained sensor sensitivities in tension in most of previous studies are higher than those of sensors using SWNTs or of traditional metal-foil strain gauges except for [32]. For instance, the identified sensor sensitivity of MWNT nanocomposite sensors is 7.0, *i.e.*, around 3.5 times higher than that of traditional metal-foil strain gauges in [17]. In [18], in the linear response region, the gauge factor is around 1.6 for 1.44 vol.% MWNT loading, and in the nonlinear region the gauge factor is around 50. The obtained sensor gauge factor is around 15 for 1.0 wt.% MWNT loading in [19]. In [21], the obtained gauge factor of the sensor with 1.0 wt.% MWNT loading is about 16. The gauge factor ranges from 3.4 to 4.3 for 0.1 wt.% MWNT content in [23]. In [24], the best sensitivities for the lowest MWNT loading of 17.0 wt.%, and for the lowest carbon nanofibers loading of 36.6 wt.% are 1.57 and 7.98, respectively. The best gauge factors are between 2.0 and 2.5 when the carbon nanofiber loading is near the percolation threshold being smaller than 0.5 vol.% in [26]. In [27], the best gauge factors obtained at ±6,000 με strain level are 22.4 in tension and 7.0 in compression for 1.0 wt.% MWNT loading. In [31], the highest sensor gauge factor is 2.78 for 0.5 wt.% MWNT loading with one direction alignment. In [32], the best sensor gauge factor is around 0.74 for 0.5 wt.% MWNT loading. For carbon nano-blacks, it was found in [23] that compared with MWNT nanocomposites, the carbon nano-blacks nanocomposites reveal a higher sensitivity to mechanical deformation. Moreover, the sensitivity of graphene/epoxy sensors was reported to be higher than those of SWNT/PMMA and MWNT/epoxy sensors [35].

As for the working mechanisms in the piezoresistive nanocomposite strain sensors, from the accumulated knowledge until now, the piezoresistivity observed in this kind of strain sensors made from CNT/polymer nanocomposites can be mainly attributed to the following three aspects:

- significant variation of conductive networks formed by CNTs, e.g., loss of contact among CNTs [18,19];
- tunneling resistance change in neighboring CNTs due to distance change [18,21,23,27,28,30,34];
- piezoresistivity of CNTs themselves due to their deformation [12,15,17].

In general, the different working mechanisms certainly result in the different sensor behaviors observed in experiments. In the previous studies of the present authors [21,27,30], by fabricating MWNT/nanocomposite sensors, we have systematically explore the influences of different fabrication conditions, type of MWNTs, *etc.* to uncover the working mechanisms of the sensors. The evidences provided in our experiments associated with the above working mechanisms may be able to reasonably explain the main trends of the different sensor piezoresistive behaviors. Here, we report some main outcomes in [21,27,30].

**Figure 9.**
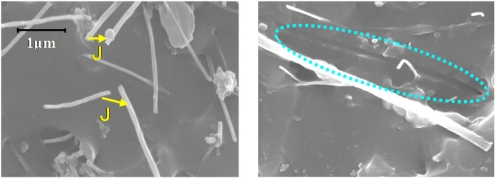
SEM images of possible tunneling effect among CNTs and evidence of weak interface between polymer and CNTs in nanocomposites.

In [21], we described the influence of tunneling effect among neighboring MWNTs. As shown in Figure 9, we have experimentally identified that there are many locations where MWNTs is close to each other in a very short distance. Based on the following Simmons’s theory for tunneling resistance [80]:

(1) $$R_{\mathrm{tunnel}}=\frac{V}{\mathrm{AJ}}=\frac{h^{2}d}{{Ae}^{2}\sqrt{2m\lambda }}\text{exp}\left(\frac{4\pi d}{h}\sqrt{2m\lambda }\right)$$

where *J* is tunneling current density, *V* the electrical potential difference, *e* the quantum of electricity, *m* the mass of electron, *h* Plank’s constant, *d* the distance between MWNTs, *λ* the height of barrier (for epoxy, 0.5 eV∼2.5 eV), and *A* the cross sectional area of tunnel (the cross sectional area of MWNT is approximately used here), it may be estimated that the tunneling resistance among CNTs increases nonlinearly, which results in a nonlinear sensor piezoresistivity. Moreover, as shown in Figure 9, very limited deformation is expected in the CNTs due to the poor stress transfer from the polymer matrix to these tubes, caused not only by the large elastic mismatch between the CNTs and the polymer but also by the weak interface strength. Therefore, the contribution of piezoresistivity of CNTs themselves to the total piezoresistivity of nanocomposite sensors may be expected to be very small.

**Figure 10.**
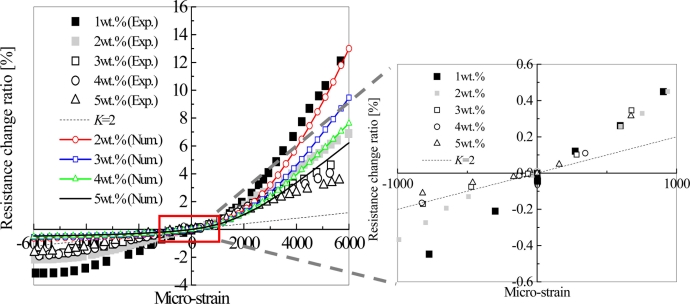
Comparison of sensor piezoresistivity for various MWNT loadings.

In [27], by investigating the influences of various parameters, *i.e.*, MWNT loading, curing temperature and mixing speed in the fabrication process, we have clearly identified the influence of the first working mechanism stated previously, *i.e.*, the change of conductive network formed by CNTs due to applied strain on the sensors. The results are shown in Figure 10.

**Figure 11.**
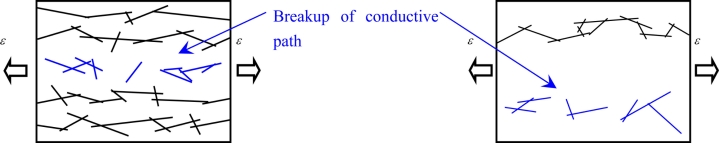
Comparison of intensive and sparse conductive networks under straining.

For comparison, the response of the conventional strain gauge is also illustrated, *i.e.*, *K* = 2. Due to this working mechanism, the sensor piezoresistivity should behave linearly since the performance of resistor network formed by CNTs is linear [73,74]. However, this change usually happens at the initial stage of straining process. That is why the linear piezoresistivity (Figure 10) was observed within a small strain range in most of previous studies [12,13,15,18,27,30]. Moreover, with the decrease of MWNT loading, the sensor sensitivity increases in Figure 10. Although this behavior may be explained from the tunneling effect as stated later, from another viewpoint, as shown in Figure 11, for an intensive conductive network with a high CNT loading, if one conductive path is broken down, the total nanocomposite resistance shows a minor variation. However, for a sparse conductive network with a very low CNT loading, for a special case of only two conductive paths in Figure 11, Δ*R* / *R*_0_ is at least around 50%, which, therefore, leads to a higher sensitivity as identified in many previous studies [15,18,19,21–24,26–28,30,31,34]. The only exception is the work of [20], which reported that the increase in CNT loading allows the SWNT-PSS/PVA film sensor to be more sensitive to strain due to creation of more nanotube-to-nanotube junctions.

**Figure 12.**
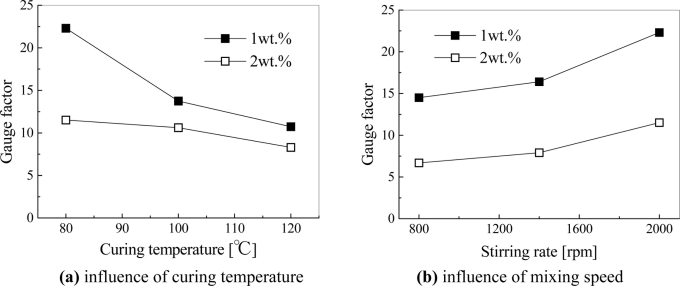
Influences of mixing speed and curing temperature on sensor sensitivity.

**Figure 13.**
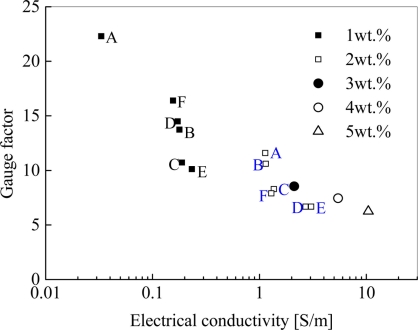
Relationship between electrical conductivity and sensor sensitivity.

In [27], we further explored the influences of various fabrication conditions, such as curing temperature and mixing speed. As shown in Figure 12, we have experimentally identified that a low curing temperature and a high mixing speed can result into higher sensor sensitivities. These conclusions are just reasonably related to some important conclusions about the electrical conductivity of nanocomposites described in Section 2 and Figure 11. In Section 2, it was stated that a low temperature in the curing process can increase the electrical resistance of nanocomposites since a sparse macroscopic conducting network may be formed by decreasing mobility of CNTs in the resulted accelerated diffusion process. Moreover, too high shear forces and too long mixing time may break up the formed networks of MWNTs, which lead to the higher resistance. Therefore, a sparse conductive network with high resistance (see Figure 11) may be favorable for obtaining a higher sensor sensitivity. For various samples under the different fabrication conditions (e.g., A–E) in [27], the relationship between the electrical conductivity of nanocomposites and sensor sensitivity is shown in Figure 13. From this figure, it is unambiguous that the samples of the higher resistances possess the higher sensor sensitivities.

**Figure 14.**
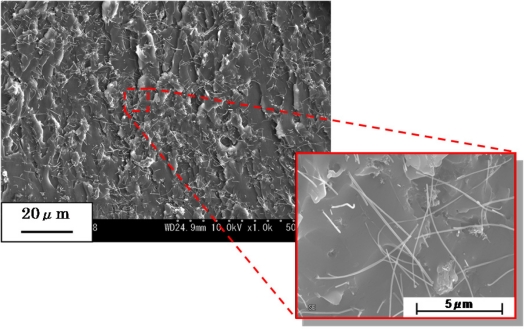
SEM images of MWNT-7/epoxy nanocomposite (2.0 wt.%).

In [30], we have systematically investigated the influences of two typical MWNTs, e.g., MWNT-7 and LMWNT-10, on sensor static and dynamic piezoresistivities. Firstly, MWNT-7 is of a large diameter (around 65 nm), and comparatively straight shape as shown in Figure 14. Therefore, it is comparatively easier to disperse it into the epoxy matrix with a quite good dispersion state (Figure 14).

**Figure 15.**
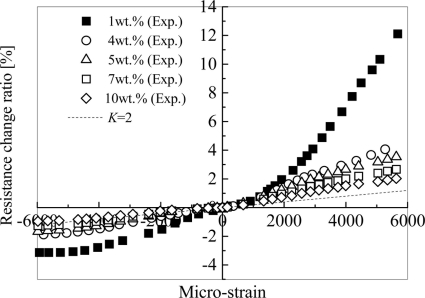
Piezoresistivity of sensor using MWNT-7.

**Figure 16.**
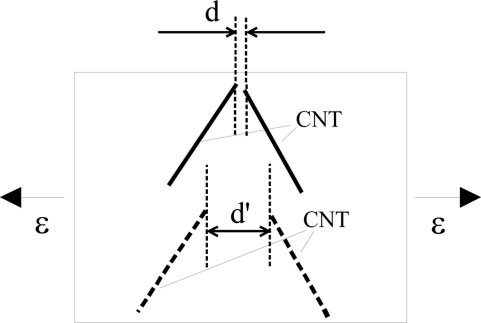
Schematic view of the working mechanism of MWNT-7/epoxy sensor.

The obtained piezoresistivities for MWNT-7/epoxy nanocomposite sensors are shown in Figure 15, which is basically similar to Figure 10 with some new data. As shown in Figure 16, it was predicted that the key working mechanism of MWNT-7/epoxy sensors may be tunneling effects among MWNTs [30]. When two CNTs are close to each other, *i.e.*, within 1.0 nm, the tunneling effects become very important, which can transfer the electrical charges between the two CNTs. The tunneling resistance between two neighboring CNTs is related to the shortest distance *d* of two CNTs as *R_tunnel_* ∝ *d* exp(*c* × *d*) [see Equation (1)], in which *c* is a constant.

Furthermore, as shown in Figure 14, some voids can be also observed on the fracture surface of MWNT-7/epoxy nanocomposite, corresponding to the locations where the CNTs are completely pulled out, indicating a weak interface between the CNTs and the epoxy matrix. Moreover, an ultrasonic testing shows that there is no apparent increase tendency in the slightly scattered values of Young’s modulus of nanocomposites with weight fractions, such as 5 wt.% of MWNT-7, which implies that the interfaces between CNTs and matrix may be weak. It means that the load-transfer ability between the matrix and MWNT-7 is very weak. Therefore, the deformation of MWNT-7 of a large diameter is very small and can be neglected. Figure 16 illustrates the tunneling effects between two CNTs in the sensor of MWNT-7/epoxy. When the distance between the CNTs increases gradually, *i.e.*, from *d* to *d*’ in Figure 16, due to the applied strain, the tunneling resistance *R_tunnel_* as shown previously will increase significantly in a nonlinear form. Therefore, the total resistance of the sensor will increase nonlinearly. Naturally, another working mechanism, *i.e.*, loss of contact among CNTs or breakup of conductive paths of CNTs must play a very important role. However, it may mainly work under the small strains [21,25]. The above working mechanism, *i.e.*, tunneling effects, may reasonably explain all behaviors of MWNT-7 sensor described previously. For instance, the nonlinear piezoresistivity behaviors of this sensor in Figure 15 should be caused by the nonlinear relationship between the distance *d* and the tunneling resistance *R_tunnel_*. Moreover, generally, a sensor is expected to work more efficiently when subjected to tensile strain as the increase of distances between neighboring CNTs is unlimited in this case. However, under compressive strains, there is a minimum distance among the CNTs due to the physical non-penetration restriction. As a result, with increase of compressive strain, the sensor sensitivity decreases and finally saturates as shown in Figure 15. Here it is worth mentioning that the MWNT-7/polymer composites may suffer from the hysteresis response as confirmed in the dynamic measurements [30], which leads to the problem of sensing repeatability. For this issue which is not the focus of the present review, our recent unpublished experimental work have identified that after the first usage of MWNT-7/epoxy or VGCF/epoxy sensors at a certain level of tensile strain, e.g., +6,000 με, the sensor repeatability can be kept very well even after 300 hundreds tests under tensile strains of +6,000 με.

**Figure 17.**
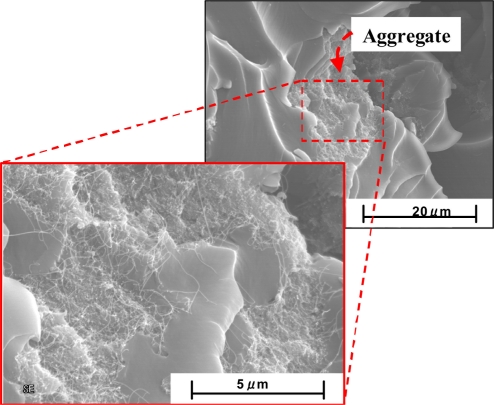
SEM images of LMWNT-10/epoxy nanocomposite (2.0 wt.%).

On the other hand, when using LMWNT-10, whose diameter is very small, *i.e.*, smaller than 10 nm, as shown in Figure 17 for LMWNT-10/epoxy nanocomposite, it can be found that there are a lot of intensive aggregates induced by strong adhesive van der Waals forces due to much smaller sizes of LMWNT-10. LMWNT-10 tubes appear to be seriously curved. The much higher resistance for LMWNT-10/epoxy nanocomposite compared with that of MWNT-7/epoxy nanocomposite confirmed the existence of these aggregates [30].

**Figure 18.**
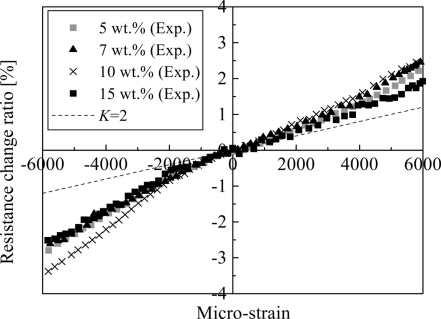
Piezoresistivity of sensor using LMWNT-10.

In Figure 18, it is shown that the piezoresistivity of LMWNT-10/epoxy sensor is approximately linear and anti-symmetric about the origin (zero strain) when subjected to tensile and compressive loadings. The LMWNT-10 weight loading has no significant influence on the sensor sensitivity or gauge factor. The gauge factors of the LMWNT-10/epoxy calibrated at ±6,000 με are only around 2 times higher than that of conventional strain gauge, *i.e.*, *K* = 2.

**Figure 19.**
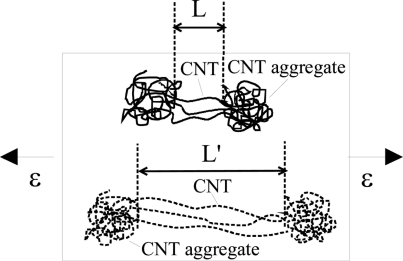
Schematic view of the working mechanism of LMWNT-10/epoxy sensor.

For the working mechanism of the LMWNT-10/epoxy sensor, as shown in Figure 19, among some intensive aggregates, there should be a few CNTs to connect them to form some complete conductive paths between the two sides of the nanocomposite at least when LWMNT-10 loading is over 5 wt.%. Otherwise, the nanocomposite should be insulating due to lack of complete paths. Naturally, the number for these bridging CNTs among aggregates should be small due to the very low electrical conductivities for LMWNT-10/epoxy nanocomposite.

When subjected to applied strains, these bridging CNTs will elongate (e.g., from *L* to *L*’ in Figure 19 after elongation) or contract depending on the tensile or compressive strains. Therefore, as reported in many studies [25,81,82], the resistance of CNTs themselves will linearly change, which leads to the piezoresistivity of CNTs and consequent nanocomposites. Based on the above stated working mechanism for the LMWNT-10/epoxy sensor, *i.e.*, the piezoresistivity of CNTs, the previously described experimental results can be partially explained. For instance, the approximate linear piezoresistivity of the LMWNT-10/epoxy sensor appears in Figure 18, which can be confirmed from the linear piezoresistivity of CNTs, as reported from many previous studies, e.g., in [25,81,82] due to the linear relationship between band-gap and small axial strain in, such as zigzag SWNTs. Also, the anti-symmetric behaviors of the LMWNT-10/epoxy sensor about the origin can be explained from the inherent anti-symmetric piezoresistivity of CNTs under small tensile and compressive strains [81]. Naturally, similar to that of MWNT-7/epoxy sensor, in this case, the breakup of conductive paths of CNTs may be considered as another mechanism which also causes the linear variation of nanocomposite resistance.

**Figure 20.**
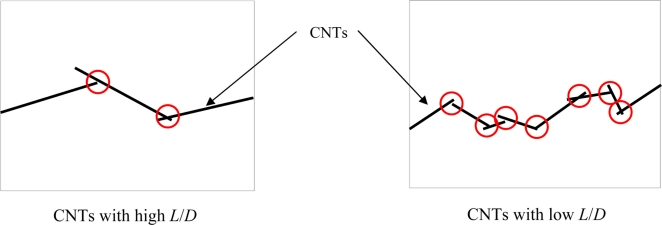
Influence of aspect ratio of fillers on conductive network.

In the above content, we have clarified the different piezoresistive behaviors of nanocomposite sensors made from two typical CNTs of the much different sizes, *i.e.*, MWNT-7 and LMWNT-10 (or SMWNT). For those filler particles, such as MWNTs or carbon nanofibers (e.g., VGCFs), which are of comparatively large sizes, straight shape, and good dispersion states in a matrix, the influence of another very important parameter, *i.e.*, aspect ratio (*L*/*D*), on sensor sensitivity, is also a very important issue. Unfortunately, up to date, there has been no reliable experimental result to clearly clarify this issue although a high *L*/*D* may decrease the percolation threshold (e.g., *ϕ_c_* = (*L*/*D*)^−1.1 ± 0.03^) and increase the electrical conductivity of nanocomposites (e.g., *σ_com_* = *σ_CNT_* ·10^0.85{log(^*^L/D^*^)–1}^·{*ϕ* – *ϕ_c_*}*^t^*) as described in Section 2. The reason may be from that the data of *L*/*D* of various CNTs of the different makers are not so clear and reliable. The provided *L*/*D* usually is not a fixed one and varies in a very wide range. However, by observing Figure 20, we may estimate the different conductive networks formed by the CNTs of the different aspect ratios.

In Figure 20, it can be found that in a complete electrical conductive path formed by a CNT of a low aspect ratio, there should be much more junction or contacting points compared with a conductive path constructed by a CNT of a high aspect ratio. Therefore, the probability of breakup of this path or happening of tunneling effect for the path containing the CNT of a low aspect ratio should be higher than that of the CNT of a high aspect ratio. Consequently, it can be estimated that the nanocomposite sensor made from the CNT of a low aspect ratio can possess a higher sensor sensitivity and low electrical conductivity compared with that made from the CNT of a high aspect ratio. For this issue, our recent unpublished work by employing MWNT-7 (diameter: 65 nm, length: 10∼30 μm, aspect ratio: >100) and VGCF (diameter: 150 nm, length: <10 μm, aspect ratio: <100) has shown that, at +6,000 με tensile strain, the sensitivity of MWNT-7/epoxy sensor is around 8.6 at 3.0 wt.% MWNT loading, which is much smaller than that of VGCF/epoxy sensor, *i.e.*, 43.01 at the same loading of VGCF. This experimental result may partially support the above estimation. However, some further experimental evidences are still needed.

### 3.2. Numerical Investigations

Compared with the above described numerous experimental investigations, unfortunately, there have been very limited explorations on the piezoresistive behavior of the CNTs filled nanocomposites based on theoretical or numerical models. Up to date, only a few studies [25,28,29] have made such an effort to try to clarify the working mechanisms and their impacts on the sensor piezoresistive behavior and sensor sensitivity. In [25], the authors employed multi-scale models to analyze this problem. Firstly, they obtained the band-gap change of SWNTs under mechanical strain. Similar to some other studies [81,82], their conclusion is that for armchair SWNTs there is no piezoresistivity, and for zigzag SWNTs [e.g., SWNT(8,0)], the resistance change is around −7.0% for 1.0% axial strain. A similar conclusion for zigzag SWNT [e.g., SWNT(12,0)] was obtained in [82], *i.e.*, 6.4% resistance change for 1.0% axial strain. In [25], they further analyzed the CNT deformation on the percolation network. It was found that within the strain range of 3.0% in CNT-polymer systems, the effect of strain on percolation appears to be negligible. This leaves the piezoresistive response of CNTs themselves, including the intertube tunneling effect, as the dominant mechanism affecting the piezoresistive response of the nanocomposite sensors. In [25], the piezoresistive response of CNTs themselves was concluded as the most important mechanism. In [28], by employing a circuit model with randomly placed resistance elements (e.g., VGCFs), which consider the contribution of tunneling resistances, the most important working mechanism was attributed to the tunneling effects among VGCFs by comparison with the experimental results.

**Figure 21.**
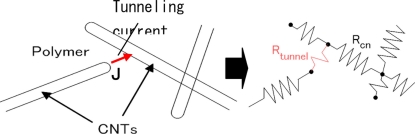
Modeling of tunneling resistance in a resistor network.

In our previous work [21], by employing the Simmons’s theory for tunneling resistance [80], *i.e.*, Equation (1), which was inserted into the 3D resistor-network model for those MWNTs of sufficiently short distances (Figure 21), we quantitatively evaluated the influence of tunneling conductivity on the total electrical conductivity of MWNT/epoxy nanocomposites.

**Figure 22.**
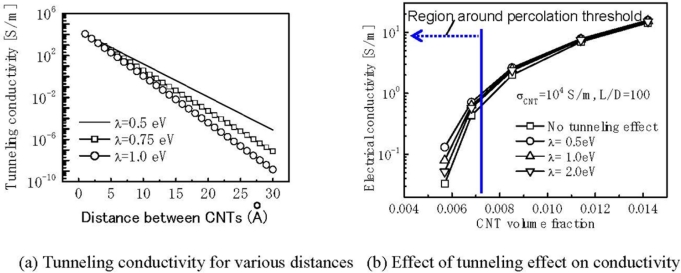
Tunneling conductivity between two CNTs, and tunneling effects on electrical conductivity of nanocomposites.

Firstly, by using Equation (1), we have evaluated the tunneling conductivity corresponding to various distances between two CNTs and various *λ* as shown in Figure 22(a). From this figure, the range for the effective tunneling effects, or cut-off distance was found to be 1.0 nm in this study, which is related to a very low tunneling conductivity [lower than 10 S/m in Figure 22(a)] compared with that of CNTs, *i.e.*, 10^4^ S/m used in [21]. Secondly, as shown in Equation (1), the tunneling resistance exponentially depends on the distance between two CNTs, which can explain the nonlinear piezoresistivity in most of studies [18,19,21–24,26–28,30–32,34]. Moreover, as shown in Figure 22(b), the significant tunneling effect can be identified by the increase of electrical conductivity when the volume fractions of CNT are near the percolation threshold of nanocomposites.

The percolation threshold is around 0.6165 vol.% obtained from the statistical percolation model for CNTs of the aspect ratio of 100 as given by *ϕ_c_* = (*L*/*D*)^−1.1±0.03^ [71]. The tunneling effect disappears gradually with increasing the amount of added CNTs. This result implies that a high sensor sensitivity in strain measurement may be achieved in a nanocomposite with the managed CNT loading being close to the percolation threshold, as identified by many previous experimental investigations [15,18,19,21–24,26–28,30,31,34], except for [20]. Naturally, for this issue, although the sensitivity would be maximized around the percolation threshold, it should be noted that the dynamic range would be relatively shortened, and the data scattering of the sensors even with the same fabrication conditions are significant. Therefore, in general, to keep a compromise between the sensor sensitivity and the reliable sensor performance, it is better to adjust the CNT loading to a level where a stable electrical conductivity of nanocomposites starts to appear. Moreover, note that for this working mechanism, *i.e.*, tunneling effect, the CNTs exhibit their own advantage over other traditional filler particles, such as CSFs, since a lot of possible locations among CNTs within a very short distance (e.g., 1.0 nm), for triggering the electrical charge transporting among CNTs, can be created by dispersing such super fine CNTs from the aspect of statistics probability. For the inconvenience caused by the nonlinear piezoresistivity, as pointed out in [21], this nonlinear characteristic can be linearized by using a logarithm-logarithm plot and then the linear calibration can be simply performed.

In [29], the present authors proposed a powerful numerical approach to analyze the piezoresistivity of the nanocomposite sensors. In this approach, by considering the “hard-core” CNTs, a 3D resistor network model [21,71] was modified by adding the tunneling resistances [see Equation (1)] between the neighboring CNTs within the cut-off distance of tunneling effect, *i.e.*, 1.0 nm in [29]. Furthermore, to analyze the piezoresistivity of the nanocomposite sensor under various strain levels, this modified 3D resistor network model was further combined with a fiber reorientation model [83], which was used to track the orientation and network change of rigid-body CNTs in the nanocomposite under applied strain. For this reason, the network change due to applied strains was also effectively modeled in [29]. The piezoresistivity of CNTs themselves was neglected in [29] based on the following reasons.

- Very limited deformation is expected in CNTs due to the poor stress transfer from the polymer matrix to these tubes, caused not only by the large mismatch of Young’s modulus between the CNTs and the polymer but also by the weak interface strength. The elastic modulus of a MWNT (500 GPa∼1.0 TPa) is about 200∼300 times higher than the epoxy (2.4 GPa∼3.0 GPa). In [21], based on our experimental observations on the fracture surface of MWNT-7/epoxy nanocomposite, the complete debonding of a MWNT-7 from the polymer matrix was frequently identified (see Figure 9), indicating low interface strength in our nanocomposite. Therefore, the strain of CNTs should be much (e.g., from several times to several 10 times) smaller than that applied on nanocomposites.
- The linear resistance change of a zigzag SWNT and some other SWNTs of a special chirality is not so obvious, e.g., the piezoresistivity *P_CNT_* = 6.4% [82], and *P_CNT_*= +7.0% for SWNT(8,1) and *P_CNT_* = −7.0% for SWNT(8,0) [25] under 1.0% axial strain. For armchair SWNT, there is no piezoresistivity. For MWNTs practically used in experiments, the amount of those SWNTs with piezoresistivity as the outmost wall may be low. Moreover, for randomly dispersed CNTs in a matrix, its effective piezoresistivity is further weakened as *P_CNT_* cos^2^ *ϕ* where *ϕ* is the angle between the axial direction of CNT and the strain direction. If we further consider the aspect of “a”, for instance, 1.0% strain on nanocomposites corresponds to 0.1% strain on CNTs (*i.e.*, 10 times smaller strain happening on CNTs due to their much higher Young’s modulus), which consequently leads to a very small electrical resistance change ratio of CNTs, e.g., around 0.6∼0.8%.

The above supporting evidences are at least valid for MWNTs of comparatively large diameters, such as MWNT-7 [21,27,30] or carbon nanofibers, such as VGCFs [24,28,34]. Naturally, for SWNTs [12,13,15] or LMWNT-10 [30] of very small diameters, the situation may be different. In this case, the piezoresistivity of CNTs may play a crucial role in the macroscopic sensor piezoresistivity as experimentally identified in [30]. Finally, in [29], the combined model was employed to predict the piezoresistivity of the nanocomposite iteratively corresponding to various strain levels with the experimental verifications (see Figure 10). From this Figure, it can be seen that the present numerical simulations can qualitatively catch the main trend of the experimental results, especially under tensile strain. In [29], for those MWNTs with a large diameter, high stiffness, straight shape and less aggregates in the epoxy matrix, the contribution of tunneling effects might be important compared with those from the network change (e.g., loss of contact among CNTs) and the piezoresistivity of CNTs and. The reasons are listed as follows:

- According to Equation (1), 1Å increase of *d* (the distance between two CNTs) can lead to 10 times lower tunneling current (*ë* = 0.5 eV, *A* = π(*D*/2)^2^, and *D* = 50 nm);
- It is interesting to note that no consistent or clear resistance change can be observed in our simulation results if only the effect of the breakup of CNT conductive network is taken into account, even by increasing the applied strain up to 1.0% [29];
- As shown in Figure 10, with considering the tunneling effects, the present model can reproduce the following features in experimental results very well. For instance, the piezoresistivity increases nonlinearly. Moreover, low CNT weight fraction can increase the sensitivity of a sensor. Furthermore, the sensor sensitivity is much lower when subjected to a compressive strain than subjected to a tensile strain since under compressive strains there is a minimum distance among the CNTs due to the non-penetration restriction applied, which leads to the decrease and final saturation of sensor sensitivity in compressive strains.

Based on the verified numerical model, some key parameters, which control the piezoresistivity behavior, such as cross-sectional area of tunnel current, height of barrier, orientation of CNTs, and electrical conductivity of CNTs and other nano-scale filler particles, were systematically investigated in [29]. We briefly describe these results in the following.

Firstly, by considering Equation (1), two important parameters are chosen, *i.e.*, *ë*: the height of barrier, and *A*: the cross-sectional areas of tunnel. It was found that with the increase of *ë* or with the decrease of *A*, the piezoresistivity of the sensor increases, which corresponds to the increase of tunneling resistance by viewing Equation (1). Moreover, it was found that the influence of *ë* is much more significant than that of *A*, which highlights the importance of selection of proper polymer with a higher *ë*. It was also found that the decrease of *A* or increase of *λ* leads to an obvious increase of the total initial resistance of sensor, *i.e.*, *R*_0_ due to substantial increase of tunneling resistance in sensor.

**Figure 23.**
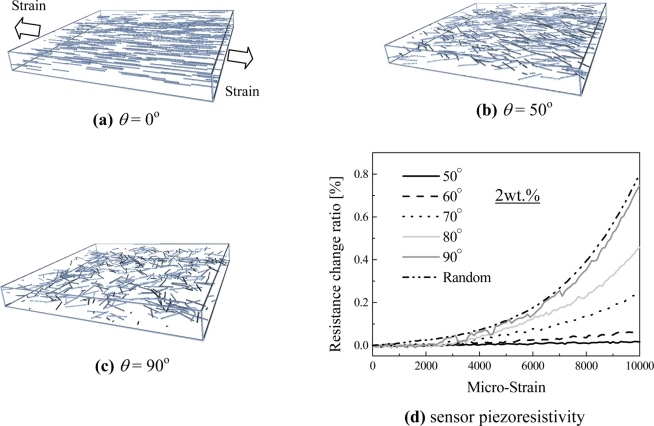
CNT alignment models and sensor piezoresistivity.

Secondly, as shown in Figure 23, we explored the influence of CNT orientations on the sensor sensitivity. In Figure 23(d), we can find that the increase of *θ* leads to a higher piezoresistivity of the sensor. It means that the state of complete randomly orientated CNTs is desirable. By observing Figure 23(a) for *θ* = 0°, we can also provide a more clear physical explanation. As shown in this figure, all CNTs are parallel to the strain direction. In this case, the distances among CNTs in the direction vertical to strain direction, which may cause the possible tunneling resistance change, do not vary significantly under tensile or compressive state. Therefore, the tunneling resistance does not change significantly in this case. Inversely, for the case of Figure 23(c), where CNTs are randomly orientated, there are a lot of possible locations where tunneling resistance can be changed due to an applied strain in any in-plane direction. Of course, in this case Figure 23(c), the total initial resistance *R*_0_ of sensor also increases as experimentally identified in [50]. Unfortunately, the above conclusion is just contrary to that obtained in [31], where the highest sensor gauge factor is 2.78 for 0.5 wt.% MWNT loading with one direction alignment. As explained in [31], the one direction alignment of CNTs along the strain direction can increase the sensor sensitivity contributed by the piezoresistivity of CNTs. The above two completely different conclusions need further substantial experimental evidences. Our previous experimental results [27] show that the higher mixing speed for dispersing CNTs into an epoxy matrix in the manufacturing process can lead to the higher sensitivity of sensor. Usually, a higher mixing speed can result in more randomly dispersed CNTs in the matrix. Therefore, this experimental evidence may partially support our numerical results in Figure 23(d). Note that to achieve a high piezoresistivity, the adjustment of above investigated factors (*A*, *λ* and *θ*) [29] always lead to the increase of the total initial resistance *R*_0_ of sensor, as experimentally identified in Figure 13.

**Figure 24.**
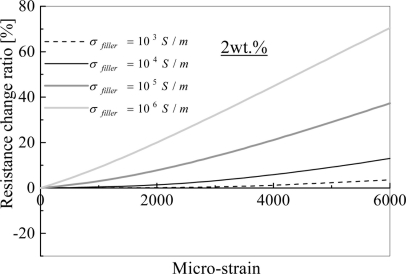
Influence of electrical conductivity of filler on sensor sensitivity.

In [29], finally, we also explored the influences of the electrical conductivity of nano-scale filler particles on sensor sensitivity. The conductivity of MWNTs was reported in a range of 5 × 10^3^∼5 × 10^6^ S/m [75,76]. Compared with MWNTs, much higher conductivities were observed in some recently developed metallic nanowires, e.g., Ag (63 × 10^6^ S/m), Cu (59.6 × 10^6^ S/m) and Au (45.2 × 10^6^ S/m). Therefore, the selection of nano-scale filler particles may play an important role in manipulating the sensor sensitivity. The numerical simulated results are shown in Figure 24. It can be seen that the resistance change ratio increases significantly with the conductivity of filler. With increase of the filler conductivity from 10^3^ S/m to 10^6^ S/m, the gauge factor corresponding to +6,000 με tensile strain increases remarkably from 6.0 to 117! In general, the conductivity of nanocomposites increases with the conductivity of filler in our previous studies [71]. Therefore, this result seems to be inconsistent to the results shown in the above content for other parameters (*A*, *ë* and *θ*), where the higher total initial resistance of nanocomposites or the higher tunneling resistance in sensors corresponds to a higher gauge factor. In fact, the overall resistance of a nanocomposite with CNT networks (Figure 11) is mainly contributed by the resistance of filler and the tunneling resistance. The decrease of filler resistance (or increase of its conductivity) leads to a higher ratio of the tunneling resistance to the overall resistance of nanocomposites. Therefore, as concluded in [29], the key to improvement of sensor sensitivity may be the increase of either tunneling resistance or the ratio of the tunneling resistance to the total resistance, rather than the total resistance itself.

## 4. Conclusions

In this review article, the piezoresistive behaviors of strain sensors made from various CNT/polymer nanocomposites have been reviewed. A lot of recent research outcomes in this field have been cited from the aspects of experiments, and numerical modeling. To understand this physical phenomenon more clearly, first we focus on the electrical percolation phenomenon of CNT/polymer nanocomposites, and electrical conductive network formed by CNTs within an insulating matrix. The influences of aspect ratio, aggregation state (or dispersion state) and curved shapes of CNTs on the percolation threshold and electrical conductivity of nanocomposites are described in detail from many experimental studies, and from a 3D statistical percolation model and a 3D resistor network model proposed by the present authors. Moreover, from the converse effects of van der Waals forces and electrostatic charges or steric effects, the influences of various fabrication conditions, *i.e.*, curing temperature, mixing speed, *etc*. on the electrical conductivity and formation of internal conductive network by CNTs, have been explained comprehensively from the experimental data of the present authors. For the piezoresistive behaviors of nanocomposite strain sensors made from various CNTs, some key features in many previous experimental studies have been described, which may be caused by the different working mechanisms in the piezoresistive nanocomposites. Furthermore, the influences of various fabrication conditions resulting in the various internal conductive network, e.g., CNTs loading, curing temperature, mixing speed and the type of CNTs on the sensor sensitivity from the present authors’ experimental investigations have been described which are tightly related to the electrical percolation phenomenon of nanocomposites. Moreover, from a powerful model proposed by the present authors to numerically simulating the macroscopic piezoresistive behaviors of nanocomposite sensors, the influences of some key factors, such as, alignment of CNTs, filler conductivity, cross-sectional area, polymer type, *etc.*, have been systematically reviewed, which can lead to some new findings to uncover the essences of various working mechanisms, and some new approaches anticipated to improve the sensor sensitivity. Naturally, the validity of these new findings and explanations needs more new experimental evidences in this field in future. From this viewpoint, many issues discussed in this review article are still unresolved ones. The previously stated three working mechanisms, *i.e.*, change of conductive networks formed by CNTs, tunneling resistance change and piezoresistivity of CNTs themselves, play different roles under the different strain levels, the different filler network morphologies, the different fillers and matrices, and some other different conditions, which finally results in the variety of the experimentally observed sensor behaviors to date.
